# Drug Quality Co-regulation Supervision Strategy Considering Collusion Behavior With New Media Participation

**DOI:** 10.3389/fpubh.2022.858705

**Published:** 2022-04-29

**Authors:** Siyi Zhang, Lilong Zhu

**Affiliations:** ^1^School of Business, Shandong Normal University, Ji'nan, China; ^2^Quality Research Center, Shandong Normal University, Ji'nan, China

**Keywords:** drug co-regulation supervision, collusion behavior, new media participation, four-party evolutionary game, simulation analysis

## Abstract

The efficiency and level of drug quality supervision are highly related to the distorted or true reporting of new media, and the collusion or non-collusion of third-party testing agencies. Therefore, based on the co-regulation information platform, considering the strategic choices of local government, drug enterprises, third-party testing agencies and new media, this article constructs a four-party evolutionary game model of co-regulation supervision. The stable equilibrium points of each participant's strategic choices are solved. The stability of the strategic combination is analyzed by *Lyapunov's* first *method*, and *Matlab 2020b* is used for simulation analysis to verify the influence of each decision variable on different players' strategic choices. The results show that, firstly, new media's true reporting can make up for the lack of supervision of drug enterprises by local government, and the greater the impact of new media reporting, the more active drug enterprises will be to produce high-quality drugs. Secondly, non-collusion of third-party testing agencies can improve the self-discipline ability of drug enterprises, encourage new media to report truthfully, and play the role of co-regulation supervision. Furthermore, the greater the probability of new media's true reporting, the more local government tend to be stricter, and the probability of strict supervision is positively related to the central government's accountability. Finally, increasing penalty for producing low-quality drugs and collusion will help standardize the behavior of drug enterprises and third-party testing agencies. This article enriches and expands the theoretical basis of the drug quality co-regulation supervision and proposes corresponding countermeasures and suggestions.

## Introduction

Drug quality supervision is an important part of ensuring the safety of public using drug and the national medical level. The development of the internet and the rise of new media have brought new opportunities and challenges to countries in drug quality supervision. In order to effectively reduce the uncertainty of regulatory decision-making, the US Food and Drug Administration utilizes new media platform, through social supervision and industry self-discipline, and after considerable development, has formed a set of effective regulatory model. The European Directorate for the Quality of Medicines is responsible for the drafting of policies on drug quality and risk prevention management of counterfeit drug, and incorporates drug safety supervision into daily management. The Pharmaceuticals and Medical Devices Agency uses non-governmental organizations, such as societies, industry associations, and the media, to supervise the self-discipline of the pharmaceutical industry, which has been an effective monitoring method for drug safety in Japan in recent years. In order to promote scientific drug supervision, the Chinese government is establishing a sound scientific and reasonable drug quality co-regulation supervision system (The above information comes from the official website of the World Health Organization and national medicine regulatory agencies of countries, such as FDA, EMA, PMDA, NMPA).

With the development of economic globalization, drug supervision is facing more problems. The development of regulatory science in the field of drug is not only between regulatory agencies and the industry, but also between patients, third-party testing agencies and society. Therefore, countries need to establish and improve drug supervision technology and information platforms, and make full use of emerging information technologies such as big data and artificial intelligence to establish drug information collection and tracking platform based on the concept of drug life cycle risk management. At the same time, there must be a standardized and authoritative new media platform to screen and release information to accelerate the development of scientific supervision and ensure the quality and safety of drug.

Therefore, this article considers new media reporting and third-party testing agency behavior, based on the co-regulation information platform, constructs an evolutionary game model involving the participation of local government, drug enterprises, third-party testing agencies and new media. The stable equilibrium point of each game player's strategic choice is solved. The stability of the strategy combination and the influence of the changes of decision variables on the strategic choice are analyzed. The following three problems are solved, firstly, how does the co-regulation system affect the strategic choices of local government, drug enterprises, third-party testing agencies and new media? Secondly, how to play the role of the co-regulation information platform to promote the drug quality co-regulation supervision? Thirdly, how does the participation of local government and new media affect the strategic choices of drug enterprises and third-party testing agencies?

The remaining parts of this article are arranged as follows. The second part combs and reviews the relevant literatures; The third part makes hypotheses and constructs an evolutionary game model which involves local government, drug enterprises, third-party testing agencies and new media; The fourth part analyzes the stability of the strategic choices of the four participants; The fifth part analyzes the stability of strategy combination according to *Lyapunov's* first *method*; The sixth part is the simulation analysis with *Matlab 2020b*; The seventh part discusses and outlines related suggestions; The last part suggests the conclusions.

## Relevant Literatures

### Drug Quality Supervision

The problems and potential hidden dangers of drug quality shouldn't be underestimated. Once a drug quality accident occurs, a strong data drive is needed to support and enhance supervision decision-making (1). The outbreak of COVID-19 has drawn drug enterprises' attention to drug quality (2). In recent years, many scholars have done a lot of research work in drug enterprise production, government supervision, third-party and new media participation, etc. (3), which provided a certain preliminary basis for the research of this article.

With the increasingly wide range of supervision channels (4), the single government supervision method (5) is not conducive to self-regulation by enterprises (6). The government should improve the supervision system, strengthen accountability (7), and play a more active role in optimizing the decision-making process (8). Establishing a digital information platform and giving economic incentives (9) can effectively promote the production of high-quality drug by drug enterprises (10).

### New Media Reporting

The emergence of new media has made the transmission of information fast and extensive (11). When new media reports on drug quality information, it can effectively assist the government in making decisions (12), and can also influence the reputation of drug enterprises through public opinion dissemination capability, thereby restricting the behavior of drug enterprises (13). However, due to the lack of effective supervision, distorted reporting by new media will cause chaos such as online trials and increase the difficulty of drug quality supervision (14). Therefore, strengthening the construction of new media platform (15) is a major challenge facing drug quality supervision in the new media environment.

### Third Party Participation

Feedback from participants outside the government can strengthen the role of a national supervision system (16). Third-party participation can promote efficient government supervision (17), which is conducive to regulating the behavior of drug enterprises (18). But the participation of third-party testing agencies in collusion will seriously affect the quality and safety of drug (19). Constructing a collusion fine mechanism (20) and strengthening the influence of a reputation mechanism can curb it (21).

In summary, there is still a lack of consideration of four participants from the perspective of co-regulation supervision. In addition, the influence of new media reporting on the different strategic choices of each participant in the co-regulation supervision is not taken into account.

Therefore, the research contributions of this article have the following three points. Firstly, considering new media participation and collusion behavior, based on the co-regulation information platform, this article builds a game model of quartet participation involving local government, drug enterprises, third-party testing agencies and new media. Secondly, considering the influence of true or distorted reports by new media, collusion or non-collusion of third-party testing agencies on the strategic choices of other participants, the evolutionary stable strategic combination under different conditions is solved. Finally, the influence of each decision variable on the strategic choices of four participants is solved and analyzed. And through *Matlab 2020b* simulation analysis, the validity of the model is verified, and countermeasures and suggestions are put forward for co-regulation supervision.

## Model Hypotheses and Construction

**Figure 1:**
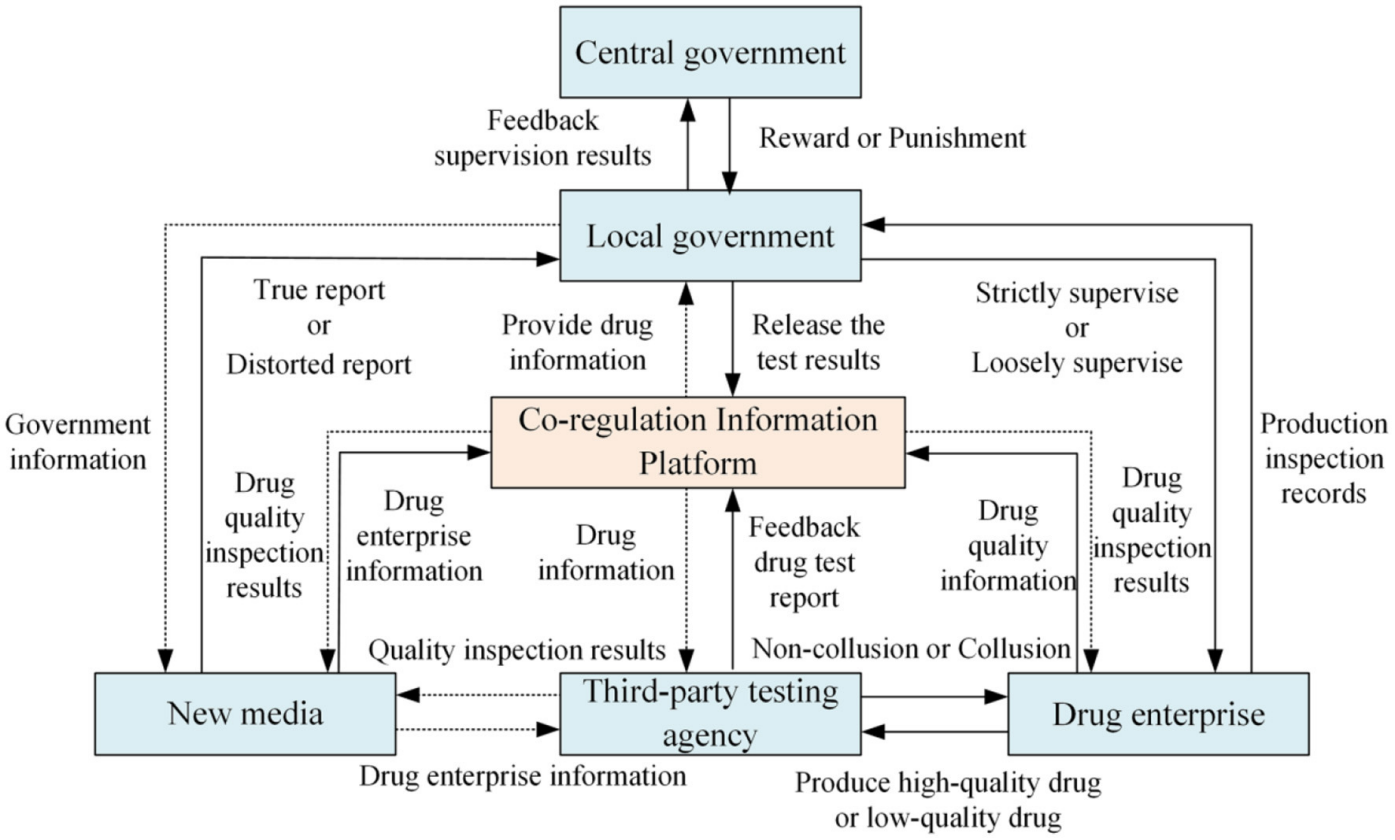
Four-party evolutionary game structure relationship. Figure is the structural relationship diagram that shows the relationship among local government, drug enterprises, third-party testing agencies and new media based on the co-regulation information platform under the reward and punishment of the central government.

This article chooses evolutionary game as the research method because it can explain the dynamic process of the evolution of each stakeholder's strategic choice and explain why this state has been reached and how to reach. The analysis paradigm of evolutionary game has a wide range of applications in social governance. Using the evolutionary perspective can more clearly see the final optimal decision of all parties in the game. Of course, when building a game model, the assumptions made for various relationships often deviate slightly from the reality, and need to be considered repeatedly, not only to meet the actual situation, but also to ensure the feasibility of the model. Therefore, considering new media participation and third-party testing agencies' collusion behavior, this article constructs the drug quality supervision structure relationship involving local government, drug enterprises, third-party testing agencies, and new media, as shown in Figure 1.

### Model Hypotheses

*H1* the local government chooses strict supervision with probability *x*, and loose supervision with probability 1 − *x*; drug enterprise chooses to produce high-quality drug with probability *y*, and low-quality drug with probability 1 − *y*; third-party testing agency chooses non-collusion with probability *z*, and collusion with probability 1 − *z*; new media chooses true reporting with probability *p*, and distorted reporting with probability 1 − *p*;*x, y, z, p* ∈ [0, 1]. The four players are bounded rational participants.

*H2* the income from the operation of drug enterprises is *R*_*e*_, the cost of producing high-quality drugs is *C*_*h*_, and the cost of producing low-quality drugs is *C*_*l*_(*C*_*l*_ < *C*_*h*_). The cost of seeking collusion is B.

*H3* the cost of strict supervision is *G*_*h*_, and the cost of loose supervision is *G*_*l*_ (*G*_*l*_ < *G*_*h*_). When local government strictly supervises, the drug enterprises that produce low-quality drugs will be fined as *F*_*e*_; the third-party testing agencies that participate in collusion will also be fined as *F*_*p*_.

*H4* the income of third-party testing agencies is *V*, and their detection cost is *C*_*p*_. If third-party testing agencies choose collusion, they need to forge test records and issue false test reports. Let the speculative cost of collusion be *C*_*t*_ (*C*_*t*_ < *B*).

*H5* the cost of new media reporting is *C*_*m*_, and the revenue from the true reporting is *R*_*ml*_. In reality, new media will distort reports in order to pursue economic benefits and obtain additional page views. Distorted reports will obtain a profit of *R*_*mh*_. According to the “spiral effect” of media communication, negative news has a magnifying effect. The news that drug enterprises produce low-quality drug will get more clicks and benefits than news that produce high-quality drug, so there are *R*_*ml*_ < *R*_*mh*_. When drug enterprises produce low-quality drug, local government loosely supervises but third-party testing agencies are not colluding, the true reports of new media will cause local government to be held accountable by the central government, setting the accountability as *F*_*g*_.

*H6* new media reports will affect the reputation of drug enterprises, which will bring economic gains or losses to them. Set the influence effect of new media reports as δ, δ ∈ [0, 1]. When drug enterprises produce high-quality drug, new media true reporting will bring a reputation value to them δ*I*, and distorted reports will cause negative impacts and economic losses δ*L*. At this time, drug enterprises can sue new media for rights protection and receive compensation *F*_*m*_. When drug enterprises produce low-quality drug, new media reporting will damage the reputation of drug enterprises and cause loss of reputation value δ*N*.

The parameters and descriptions of this article are shown in Table 1.

**Table 1:** Related parameter description.

**Parameter**	**Description**
*x*	Probability of local government choosing strict supervision
*y*	Probability of drug enterprises producing high-quality drug
*z*	Probability of non-collusion of third-party testing agencies
*p*	Probability of true reporting of new media
*R*_*e*_	Income of drug enterprises
*C*_*h*_	The cost of drug enterprises producing high-quality drug
*C*_*l*_	The cost of drug enterprises producing low-quality drug
*B*	The cost of drug enterprises seeking collusion
*G*_*h*_	The cost of strict supervision of local government
*G*_*l*_	The cost of loose supervision of local government
*F*_*e*_	Fines for producing low-quality drug of drug enterprises
*F*_*p*_	Fines for collusion of third-party testing agencies
*V*	Testing income of third-party testing agencies
*C*_*p*_	Detection cost of third-party testing agencies
*C*_*t*_	Speculation cost of third-party testing agencies
*C*_*m*_	The cost of new media reporting
*R*_*ml*_	The revenue from the true reporting of the new media
*R*_*mh*_	The revenue from the distorted reporting of the new media
*F*_*g*_	Punishment for loose supervision of local government
δ	The influence effect of new media reports
*I*	The reputation value of drug enterprises producing high-quality drug
*L*	Economic loss caused to drug enterprises by new media distorted reports
*F*_*m*_	Compensation obtained by drug enterprises suing new media
*N*	Loss of reputation of drug enterprises producing low-quality drug

### Model Construction

Based on the above hypotheses, this article constructs a four-party mixed strategy game matrix for local government, drug enterprises, third-party testing agencies and new media, as shown in Table 2.

**Table 2:** Four-party mixed strategy game matrix.

**Choice of strategy**		**Third-party testing agencies**		**Local government**			
				**Strictly supervise *x***		**Loosely supervise 1 − *x***	
			**New media**	**True report p**	**Distorted report 1 − *p***	**True report p**	**Distorted report 1 − *p***
Drug enterprises	Produce high-quality drug *y*	Non-collusion *z*		*R*_*e*_ − *C*_*h*_ + δ*I* *V* − *C*_*p*_ *R*_*ml*_ − *C*_*m*_ −*G*_*h*_	*R*_*e*_ − *C*_*h*_ − δ*L* + *F*_*m*_ *V* − *C*_*p*_ *R*_*mh*_ − *C*_*m*_ − *F*_*m*_ −*G*_*h*_	*R*_*e*_ − *C*_*h*_ + δ*I* *V* − *C*_*p*_ *R*_*ml*_ − *C*_*m*_ −*G*_*l*_	*R*_*e*_ − *C*_*h*_ − δ*L* + *F*_*m*_ *V* − *C*_*p*_ *R*_*mh*_ − *C*_*m*_ − *F*_*m*_ −*G*_*l*_
		Collusion 1 − *z*		*R*_*e*_ − *C*_*h*_ + δ*I* *V* − *C*_*p*_ *R*_*ml*_ − *C*_*m*_ −*G*_*h*_	*R*_*e*_ − *C*_*h*_ − δ*L* + *F*_*m*_ *V* − *C*_*p*_ *R*_*mh*_ − *C*_*m*_ − *F*_*m*_ −*G*_*h*_	*R*_*e*_ − *C*_*h*_ + δ*I* *V* − *C*_*p*_ *R*_*ml*_ − *C*_*m*_ −*G*_*l*_	*R*_*e*_ − *C*_*h*_ − δ*L* + *F*_*m*_ *V* − *C*_*p*_ *R*_*mh*_ − *C*_*m*_ − *F*_*m*_ −*G*_*l*_
	Produce low-quality drug 1 − *y*	Non-collusion *z*		*R*_*e*_ − *C*_*l*_ − δ*N* − *F*_*e*_ *V* − *C*_*p*_ *R*_*mh*_ − *C*_*m*_ *F*_*e*_ − *G*_*h*_	*R*_*e*_ − *C*_*l*_ − δ*N* − *F*_*e*_ *V* − *C*_*p*_ *R*_*mh*_ − *C*_*m*_ *F*_*e*_ − *G*_*h*_	*R*_*e*_ − *C*_*l*_ − δ*N* − *F*_*e*_ *V* − *C*_*p*_ *R*_*mh*_ − *C*_*m*_ *F*_*e*_ − *G*_*l*_ − *F*_*g*_	*R*_*e*_ − *C*_*l*_ − δ*N* − *F*_*e*_ *V* − *C*_*p*_ *R*_*mh*_ − *C*_*m*_ *F*_*e*_ − *G*_*l*_ − *F*_*g*_
		Collusion 1 − *z*		*R*_*e*_ − *C*_*l*_ − δ*N* − *F*_*e*_ − *B* *V* − *C*_*p*_ + *B* − *C*_*t*_ − *F*_*p*_ *R*_*mh*_ − *C*_*m*_ *F*_*e*_ + *F*_*p*_ − *G*_*h*_	*R*_*e*_ − *C*_*l*_ − δ*N* − *F*_*e*_ − *B* *V* − *C*_*p*_ + *B* − *C*_*t*_ − *F*_*p*_ *R*_*mh*_ − *C*_*m*_ *F*_*e*_ + *F*_*p*_ − *G*_*h*_	*R*_*e*_ − *C*_*l*_ − δ*I* − *B* *V* − *C*_*p*_ + *B* − *C*_*t*_ *R*_*ml*_ − *C*_*m*_ −*G*_*l*_	*R*_*e*_ − *C*_*l*_ − δ*N* − *F*_*e*_ − *B* *V* − *C*_*p*_ + *B* − *C*_*t*_ − *F*_*p*_ *R*_*mh*_ − *C*_*m*_ *F*_*e*_ + *F*_*p*_ − *F*_*g*_ − *G*_*l*_

## Analysis of the Strategic Choice Stability

### The Local Government's Strategic Choice Stability

The expected benefit of local government choosing the “strict supervision” strategy is:


(1) $$\begin{array}{l} E_{x}=y\left(-G_{h}\right)+\left(1-y\right)z\left(F_{e}-G_{h}\right)\\ \;+\left(1-y\right)\left(1-z\right)\left(F_{e}+F_{p}-G_{h}\right) \end{array}$$

The expected benefit of local government choosing the “loose supervision” strategy is:


(2) $$\begin{array}{l} E_{1-x}=y\left(-G_{l}\right)+\left(1-y\right)z(F_{e}-G_{l}-F_{g}+\left(1-y\right)\left(1-z\right)p\left(-G_{l}\right)\\ \;+\left(1-y\right)\left(1-z\right)\left(1-p\right)\left(F_{e}+F_{p}-F_{g}-G_{l}\right) \end{array}$$

The replicated dynamic equation and the first derivative of the local government's strategic choice are:


(3) $$\begin{array}{l} F\left(x\right)=dx/dt=x\left(E_{x}-\overset{-}{E}\right)=x\left(1-x\right)\left(E_{x}-E_{1-x}\right)\\ =x(1-x)[G_{l}-G_{h}+F_{g}-yF_{g}\\ +(1-y)(1-z)p(F_{e}+F_{p}-F_{g})] \end{array}$$

(4) $$\begin{array}{l} F^{\prime }(x)=(1-2x)[G_{l}-G_{h}+F_{g}-yF_{g}\\ +(1-y)(1-z)p(F_{e}+F_{p}-F_{g})] \end{array}$$

According to the stability theorem of differential equations, if the probability of local government choosing the “strict supervision” strategy is to be in a stable state, the following conditions must be met: *F*(*x*) < 0 and *F*′(*x*) < 0.

**Proposition 1** When *y* < *y*_0_, *z* < *z*_0_ or *p* > *p*_0_, the local government's stabilization strategy is “strict supervision.” When *y* > *y*_0_, *z* > *z*_0_ or *p* < *p*_0_, its stabilization strategy is “loose supervision.” When *y* = *y*_0_,*z* = *z*_0_ or *p* = *p*_0_, we are unable to determine its stabilization strategy. The thresholds are


$$\begin{array}{l} y_{0}=1-\frac{G_{h}-G_{l}}{F_{g}+\left(1-z\right)p\left(F_{e}+F_{p}-F_{g}\right)},\\ z_{0}=1-\frac{G_{h}-G_{l}-F_{g}+yF_{g}}{\left(1-y\right)p\left(F_{e}+F_{p}-F_{g}\right)},\\ p_{0}=\frac{G_{h}-G_{l}-F_{g}+yF_{g}}{\left(1-y\right)\left(1-z\right)\left(F_{e}+F_{p}-F_{g}\right)}. \end{array}$$

***Proof*** Make *G*(*y, z, p*) = *G*_*l*_ − *G*_*h*_ + *F*_*g*_ − *yF*_*g*_ + (1 − *y*)(1 − *z*)*p*(*F*_*e*_ + *F*_*p*_ − *F*_*g*_), when *G*(*y, z, p*) = 0,$y_{0}=1-\frac{G_{h}-G_{l}}{F_{g}+\left(1-z\right)p\left(F_{e}+F_{p}-F_{g}\right)}$,$z_{0}=1-\frac{G_{h}-G_{l}-F_{g}+yF_{g}}{\left(1-y\right)p\left(F_{e}+F_{p}-F_{g}\right)}$,$p_{0}=\frac{G_{h}-G_{l}-F_{g}+yF_{g}}{\left(1-y\right)\left(1-z\right)\left(F_{e}+F_{p}-F_{g}\right)}$ can be calculated. Because ∂*G*(*y, z, p*)/∂*y* < 0, ∂*G*(*y, z, p*)/∂*z* < 0 and ∂*G*(*y, z, p*)/∂*p* > 0, *G*(*y, z, p*) is a decreasing function of *y* and *z*, and an increasing function of *p*. When, *z* < *z*_0_ or *p* > *p*_0_,*G*(*y, z, p*) > 0,$F^{\prime }\left(x\right){|}_{x=1}<0$ and *F*(*x*)|_*x* = 1_ = 0 can be calculated, so *x* = 1 is stable. When *y* > *y*_0_, or *p* < *p*_0_ „ $F^{\prime }\left(x\right){|}_{x=0}<0$ and *F*(*x*)|_*x* = 0_ = 0 can be calculated, so *x* = 0 is stable. When, *z* = *z*_0_ or *p* = *p*_0_,*G*(*y, z, p*) = 0 and *F*′(*x*) = 0 can be calculated, we are unable to determine a stable strategy.

Proposition 1 shows that if *y* or *z* increases, the local government's stabilization strategy will change from strict supervision to loose supervision. If increases, the local government's stabilization strategy will change from loose supervision to strict supervision. Therefore, in drug quality co-regulation supervision, true reporting from new media can urge local government to perform strict supervision duty. When drug enterprises consciously produce high-quality drugs, local government will choose loose supervision in order to maximize social benefits and save supervision cost.

In summary, the response function of *x* is


(5) $$x=\{\begin{array}{ccc} 0 & if & y>1-\frac{G_{h}-G_{l}}{F_{g}+(1-z)p(F_{e}+F_{p}-F_{g})}\\ \left[0,1\right] & if & y=1-\frac{G_{h}-G_{l}}{F_{g}+(1-z)p(F_{e}+F_{p}-F_{g})}\\ 1 & if & y<1-\frac{G_{h}-G_{l}}{F_{g}+(1-z)p(F_{e}+F_{p}-F_{g})} \end{array}$$

**Figure 2:**
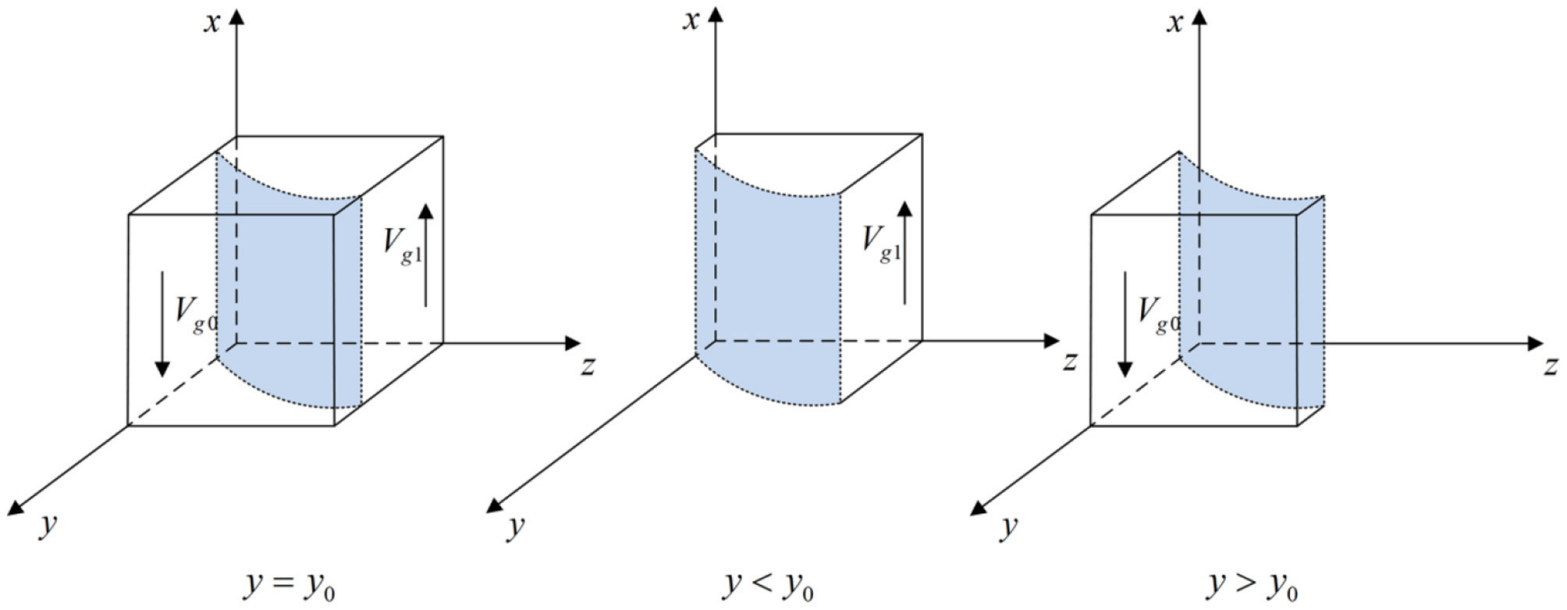
Phase diagram of the local government strategic choice. Figure is the phase diagram that shows the evolutionary trend of local government's strategy obtained by calculating the response function of the probability of local government choosing the “strict supervision” strategy.

According to Proposition 1, the phase diagram of local government strategic choice is shown in Figure 2.

It can be seen from Figure 2 that the volume of *V*_*g*1_ is the probability that local government chooses the “strict supervision” strategy, and the volume of *V*_*g*0_ is the probability that local government chooses the “loose supervision” strategy. And,


(6) $$\begin{array}{l} V_{g1}=\int_{0}^{1}\int_{0}^{1}y_{0}dzdx\\ =1-\frac{G_{h}-G_{l}}{p\left(F_{g}-F_{e}-F_{p}\right)}\ln\;\frac{F_{g}}{F_{g}-p\left(F_{g}-F_{e}-F_{p}\right)} \end{array}$$

(7) $$\begin{array}{l} V_{g0}=1-V_{g1}=\frac{G_{h}-G_{l}}{p\left(F_{g}-F_{e}-F_{p}\right)}\ln\;\frac{F_{g}}{F_{g}-p\left(F_{g}-F_{e}-F_{p}\right)} \end{array}$$

**Corollary 1.1** when local government imposes more fines on drug enterprises and third-party testing agencies, it tends more to choose the “strict supervision” strategy.

***Proof*** According to the probability *V*_*g*1_, the first-order partial derivatives of *F*_*e*_ and *F*_*p*_ can be calculated:


$$\begin{array}{l} \frac{\partial V_{g1}}{\partial F_{e}}=\frac{G_{h}-G_{l}}{\left(F_{g}-F_{e}-F_{p}\right)}[\frac{1}{F_{g}-p(F_{g}-F_{e}-F_{p})}\\ -\frac{1}{p(F_{g}-F_{e}-F_{p})}\ln\frac{F_{g}}{F_{g}-p(F_{g}-F_{e}-F_{p})}]>0\\ \frac{\partial V_{g1}}{\partial F_{p}}=\frac{G_{h}-G_{l}}{\left(F_{g}-F_{e}-F_{p}\right)}[\frac{1}{F_{g}-p(F_{g}-F_{e}-F_{p})}\\ -\frac{1}{p(F_{g}-F_{e}-F_{p})}\ln\frac{F_{g}}{F_{g}-p(F_{g}-F_{e}-F_{p})}]>0 \end{array}$$

Corollary 1.1 shows that the probability of local government choosing “strict supervision” strategy is an increasing function of *F*_*e*_ and *F*_*p*_. With the increase of *F*_*e*_ and, local government will conduct “strict supervision” in order to obtain more revenue.

**Corollary 1.2** When local government pays more additional cost for strict supervision, it tends more to choose the “loose supervision” strategy.

***Proof*** According to the probability *V*_*g*0_, the first-order partial derivatives of *G*_*h*_ − *G*_*l*_ can be calculated:

Corollary 1.2 shows that the probability of local government choosing the “loose supervision” strategy is an increasing function of *G*_*h*_ − *G*_*l*_. The higher the additional cost of strict supervision, local government will tend to choose loose supervision in order to save cost.

### Drug Enterprises' Strategic Choice Stability

The expected benefit of drug enterprises choosing the “producing high-quality drug” strategy is:


(8) $$\begin{array}{l} E_{y}=p\left[R_{e}-C_{h}+\delta I\right]+\left(1-p\right)\left(R_{e}-C_{h}-\delta L+F_{m}\right) \end{array}$$

The expected benefit of drug enterprises choosing the “producing low-quality drug” strategy is:


(9) $$\begin{array}{l} E_{1-y}=z\left(R_{e}-C_{l}-\delta N-F_{e}\right)+\left(1-z\right)\\ \left(R_{e}-C_{l}-\delta N-F_{e}-B\right)+\left(1-z\right)\left(1-x\right)p\left(\delta I+\delta N+F_{e}\right) \end{array}$$

The replicated dynamic equation and the first derivative of drug enterprises' strategic choice are:


(10) $$\begin{array}{l} F(y)=dy/dt=y(E_{y}-\bar{E})\\ =y(1-y)(E_{y}-E_{1-y})=y(1-y)[C_{l}-C_{h}+F_{m}+(1-z)B+F_{e}+\delta (N-L)\\ +p(\delta I+\delta L-F_{m})-(1-z)(1-x)p(\delta I+\delta N+F_{e})] \end{array}$$

(11) $$\begin{array}{l} F^{\prime }(y)=(1-2y)[C_{l}-C_{h}+F_{m}+(1-z)B+F_{e}+\delta (N-L)\\ +p(\delta I+\delta L-F_{m})-(1-z)(1-x)p(\delta I+\delta N+F_{e})] \end{array}$$

According to the stability theorem of differential equations, if the probability of drug enterprises choosing the “producing high-quality drug” strategy is to be in a stable state, the following conditions must be met: *F*(*y*) = 0 and *F*′(*y*) = 0.

**Proposition 2** When *x* < *X*_1_, *z* > *z*_1_ or *p* > *p*_1_, drug enterprises' stabilization strategy is “producing high-quality drug.” When *x* < *x*_1_,*z* < *z*_1_ or *p* < *p*_1_, their stabilization strategy is “producing low-quality drug.” When *x* = *x*_1_,*z* = *z*_1_ or *p* = *p*_1_, we are unable to determine their stabilization strategy. The thresholds are


$$\begin{array}{l} x_{1}=1-\frac{C_{l}-C_{h}+F_{m}+\left(1-z\right)B+F_{e}+\delta \left(N-L\right)+p\left(\delta I+\delta L-F_{m}\right)}{\left(1-z\right)p\left(\delta I+\delta N+F_{e}\right)},\\ z_{1}=1-\frac{C_{h}-C_{l}-F_{m}-F_{e}-\delta \left(N-L\right)-p\left(\delta I+\delta L-F_{m}\right)}{B-\left(1-x\right)p\left(\delta I+\delta N+F_{e}\right)},\\ p_{1}=\frac{C_{h}-C_{l}-F_{m}-F_{e}-\delta \left(N-L\right)-\left(1-z\right)B}{\delta I+\delta L-F_{m}-\left(1-z\right)\left(1-x\right)\left(\delta I+\delta N+F_{e}\right)}. \end{array}$$

***Proof*** Make, when *K*(*x, z, p*) = 0, $z_{1}=1-\frac{C_{h}-C_{l}-F_{m}-F_{e}-\delta \left(N-L\right)-p\left(\delta I+\delta L-F_{m}\right)}{B-\left(1-x\right)p\left(\delta I+\delta N+F_{e}\right)}$, $p_{1}=\frac{C_{h}-C_{l}-F_{m}-F_{e}-\delta \left(N-L\right)-\left(1-z\right)B}{\delta I+\delta L-F_{m}-\left(1-z\right)\left(1-x\right)\left(\delta I+\delta N+F_{e}\right)}$ can be calculated. Because ∂*K*(*x, z, p*)/∂*x* > 0,∂*K*(*x, z, p*)/∂*z* > 0 and ∂*K*(*x, z, p*)/∂*p* > 0, *K*(*x, z, p*) is an increasing function of *x*, *z* and *p*. When *x* > *x*_1_, *z* > *z*_1_ or *p* > *p*_1_, *K*(*x, z, p*) > 0, $F^{\prime }\left(y\right){|}_{y=1}<0$ and *F*(*y*)|_*y* = 1_ = 0 can be calculated, so *y* = 1 is stable. When *x* < *x*_1_,*z* < *z*_1_ or *p* < *p*_1_,*K*(*x, z, p*) < 0,$F^{\prime }\left(y\right){|}_{y=0}<0$ and *F*(*y*)|_*y* = 0_ = 0 can be calculated, so *y* = 0 is stable. When *x* = *x*_1_,*z* = *z*_1_ or *p* = *p*_1_,*K*(*x, z, p*) = 0 and can be calculated, we are unable to determine a stable strategy.

Proposition 2 shows that if *x*, or *p* increases, drug enterprises' stabilization strategy will change from producing low-quality drug to producing high-quality drug. Therefore, strict supervision by local government, urging third-party testing agencies to perform non-collusive social responsibility, and encouraging true reporting by new media can all prompt drug enterprises to proactively produce high-quality drug.

In summary, the response function of y is:


(12) $$y=\{\begin{array}{ccc} 0 & if & p<\frac{C_{h}-C_{l}-F_{m}-F_{e}-\delta (N-L)-(1-z)B}{\delta I+\delta L-F_{m}-(1-z)(1-x)(\delta I+\delta N+F_{e})}\\ \left[0,1\right] & if & p=\frac{C_{h}-C_{l}-F_{m}-F_{e}-\delta (N-L)-(1-z)B}{\delta I+\delta L-F_{m}-(1-z)(1-x)(\delta I+\delta N+F_{e})}\\ 1 & if & p>\frac{C_{h}-C_{l}-F_{m}-F_{e}-\delta (N-L)-(1-z)B}{\delta I+\delta L-F_{m}-(1-z)(1-x)(\delta I+\delta N+F_{e})} \end{array}$$

**Figure 3:**
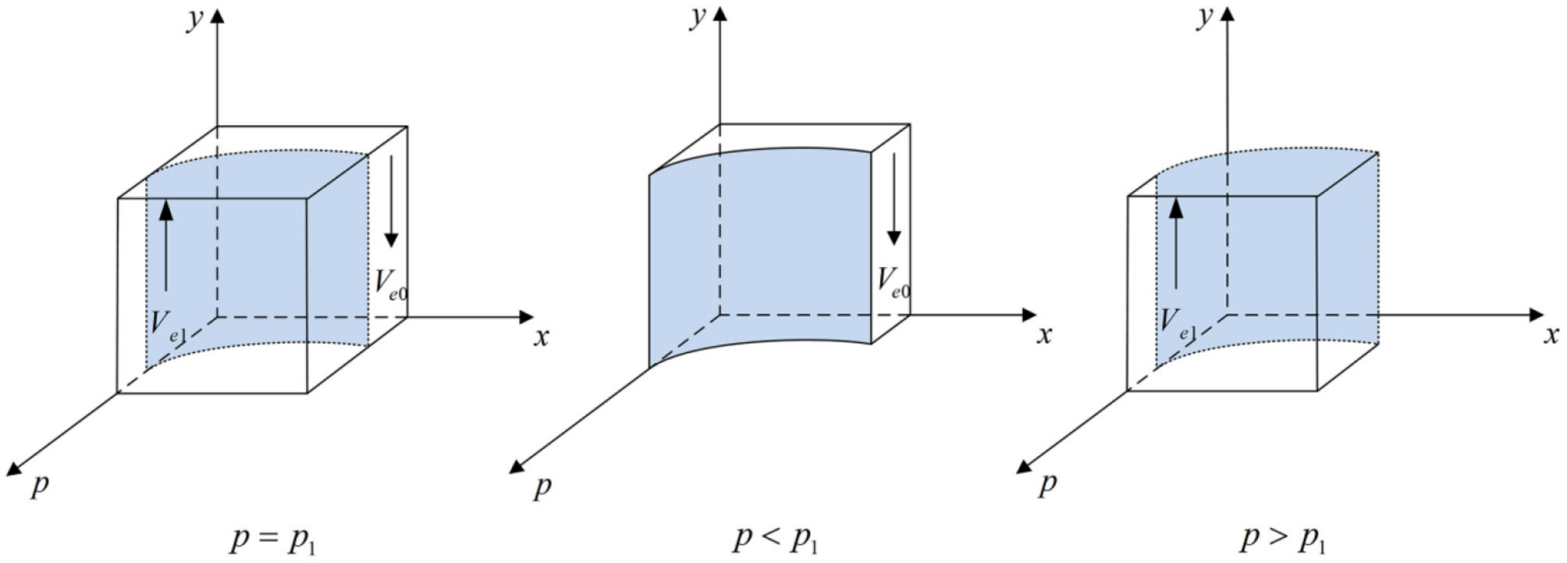
Phase diagram of drug enterprises strategic choice. Figure is the phase diagram that shows the evolutionary trend of drug enterprises' strategy obtained by calculating the response function of the probability of drug enterprises choosing the “producing high-quality drug” strategy.

According to Proposition 2, the phase diagram of drug enterprises strategic choice is shown in Figure 3.

It can be seen from Figure 3 that the volume of *V*_*e*1_ is the probability that drug enterprises choose the “producing high-quality drug” strategy, and the volume of *V*_*e*0_ is the probability that drug enterprises choose the “producing low-quality drug” strategy. And,


(13) $$\begin{array}{l} V_{e0}=\int_{0}^{1}\int_{0}^{1}p_{1}dxdy\\ =\frac{C_{h}-C_{l}-F_{m}-F_{e}-\delta \left(N-L\right)-\left(1-z\right)B}{\left(1-z\right)\left(\delta I+\delta N+F_{e}\right)}\\ \ln\;\frac{\delta I+\delta L-F_{m}}{\delta I+\delta L-F_{m}-\left(1-z\right)\left(\delta I+\delta N+F_{e}\right)} \end{array}$$

(14) $$\begin{array}{l} V_{e1}=1-V_{e0}\\ =1-\frac{C_{h}-C_{l}-F_{m}-F_{e}-\delta \left(N-L\right)-\left(1-z\right)B}{\left(1-z\right)\left(\delta I+\delta N+F_{e}\right)}\\ \ln\;\frac{\delta I+\delta L-F_{m}}{\delta I+\delta L-F_{m}-\left(1-z\right)\left(\delta I+\delta N+F_{e}\right)} \end{array}$$

**Corollary 2.1** The higher the reputation value of high-quality drugs produced by drug enterprises and the greater the reputation loss of low-quality drugs, the greater the probability of producing high-quality drugs.

***Proof*** Make *A* = *C*_*h*_ − *C*_*l*_ − *F*_*m*_ − *F*_*e*_−δ(*N* − *L*)−(1 − *z*)*B*, *T* = δ*I* + δ*N* + *F*_*e*_. According to the probability *V*_*e*1_, the first-order partial derivatives of *N* and *I* can be calculated:


(15) $$\begin{array}{l} \frac{\partial V_{e1}}{\partial N}\text{=}\frac{\delta \left(T+A\right)}{\left(1-z\right)T^{2}}\ln\;\frac{\delta I+\delta L-F_{m}}{\delta I+\delta L-F_{m}-\left(1-z\right)T}\\ -\frac{A}{T}\frac{\delta }{\delta I+\delta L-F_{m}-\left(1-z\right)T}>0 \end{array}$$

(16) $$\begin{array}{l} \frac{\partial V_{e1}}{\partial I}\text{=}\frac{\delta A}{\left(1-z\right)T^{2}}\ln\;\frac{\delta I+\delta L-F_{m}}{\delta I+\delta L-F_{m}-\left(1-z\right)T}\\ -\frac{A}{\left(1-z\right)T}\left[\frac{\delta }{\delta I+\delta L-F_{m}}-\frac{z\delta }{\delta I+\delta L-F_{m}-\left(1-z\right)T}\right]>0 \end{array}$$

Corollary 2.1 shows that increasing the price that drug enterprises need to pay for producing low-quality drugs or increasing the reputation benefits they earn from producing high-quality drugs can increase the probability that drug enterprises choose the strategy of “producing high-quality drug.”

**Corollary 2.2** The higher the additional cost of producing high-quality drug by drug enterprises, the higher the probability of producing low-quality drug; the greater the influence effect of new media reporting, the lower the probability of drug enterprises producing low-quality drug.

***Proof*** Make *A* = *C*_*h*_ − *C*_*l*_ − *F*_*m*_ − *F*_*e*_ − δ(*N* − *L*)−(1 − *z*)*B*, *T* = δ*I* + δ*N* + *F*_*e*_, *U* = δ*I*+δ*L* − *F*_*m*_. According to the probability, the first-order partial derivatives of (*C*_*h*_ − *C*_*l*_) and δ can be calculated:


(17) $$\begin{array}{l} \frac{\partial V_{e0}}{\partial \delta }\text{=}-\frac{\left(N-L\right)T+A\left(I+N\right)}{\left(1-z\right)T^{2}}\ln\;\frac{U}{U-\left(1-z\right)T}\\ -\frac{A}{\left(1-z\right)T}\left[\frac{I+L-\left(1-z\right)\left(I+N\right)}{U-\left(1-z\right)T}-\frac{I+L}{U}\right]<0 \end{array}$$

Corollary 2.2 shows that the probability *V*_*e*0_ is an increasing function of (*C*_*h*_ − *C*_*l*_), and a decreasing function of δ. When the additional cost of producing high-quality drug increases, drug enterprises tend to produce low-quality drug for economic benefits; when the influence effect of new media reporting is greater, in order to reduce reputation loss and ensure economic benefits, they are less likely to choose the strategy of “producing low-quality drug.”

### Third-Party Testing Agencies' Strategic Choice Stability

The expected benefit of third-party testing agencies choosing the “non-collusion” strategy is:


(18) $$\begin{array}{l} E_{z}=V-C_{p} \end{array}$$

The expected benefit of third-party testing agencies choosing the “collusion” strategy is:


(19) $$\begin{array}{l} E_{1-z}=\left(1-y\right)\left(V-C_{p}+B-C_{t}-F_{p}\right)+\left(1-y\right)\left(1-x\right)pF_{p} \end{array}$$

The replicated dynamic equation and the first derivative of third-party testing agencies' strategic choice are:


(20) $$\begin{array}{l} F\left(z\right)=dz/dt=z\left(E_{z}-\overset{-}{E}\right)=z\left(1-z\right)\left(E_{z}-E_{1-z}\right)\\ =z(1-z)[y(V-C_{p})+(1-y)(F_{p}+C_{t}-B)\\ -(1-y)(1-x)pF_{p}] \end{array}$$

(21) $$\begin{array}{l} F^{\prime }\left(z\right)=\left(1-2z\right)[y\left(V-C_{p}\right)+\left(1-y\right)\left(F_{p}+C_{t}-B\right)\\ -\left(1-y\right)\left(1-x\right)pF_{p}] \end{array}$$

According to the stability theorem of differential equations, if the probability of third-party testing agencies choosing the “non-collusion” strategy is to be in a stable state, the following conditions must be met: *F*(*z*) = 0 and *F*′(*z*) = 0.

**Proposition 3** When *x* > *x*_2_, or *p* < *p*_2_, third-party testing agencies' stabilization strategy is “non-collusion.” When *x* < *x*_2_,*y* < *y*_2_ or *p* > *p*_2_, their stabilization strategy is “collusion.” When *x* = *x*_2_, *y* = *y*_2_ or *p* = *p*_2_ we are unable to determine their stabilization strategy. The thresholds are $x_{2}=1-\frac{y\left(V-C_{p}\right)+\left(1-y\right)\left(F_{p}+C_{t}-B\right)}{\left(1-y\right)pF_{p}}$, $y_{2}=\frac{B-C_{t}-F_{p}+\left(1-x\right)pF_{p}}{V-C_{p}+B-C_{t}-F_{p}+\left(1-x\right)pF_{p}}$, $p_{2}=\frac{y\left(V-C_{p}\right)+\left(1-y\right)\left(F_{p}+C_{t}-B\right)}{\left(1-y\right)\left(1-x\right)F_{p}}$.

***Proof*** Make *J*(*x, y, p*) = *y*(*V* − *C*_*p*_) + (1 − *y*)(*F*_*p*_ + *C*_*t*_ − *B*) − (1 − *y*)(1 − *x*)*pF*_*p*_, when *J*(*x, y, p*) = 0,$x_{2}=1-\frac{y\left(V-C_{p}\right)+\left(1-y\right)\left(F_{p}+C_{t}-B\right)}{\left(1-y\right)pF_{p}}$,$y_{2}=\frac{B-C_{t}-F_{p}+\left(1-x\right)pF_{p}}{V-C_{p}+B-C_{t}-F_{p}+\left(1-x\right)pF_{p}}$,$p_{2}=\frac{y\left(V-C_{p}\right)+\left(1-y\right)\left(F_{p}+C_{t}-B\right)}{\left(1-y\right)\left(1-x\right)F_{p}}$ can be calculated. Because ∂*J*(*x, y, p*)/∂*x* > 0,∂*J*(*x, y, p*)/∂*y* > 0 and, *J*(*x, y, p*) is an increasing function of *x* and *y*, and a decreasing function of *p*. When *x* > *x*_2_, *y* > *y*_2_ or *p* < *p*_2_,*J*(*x, y, p*) > 0,$F^{\prime }\left(z\right){|}_{z=1}<0$ and *F*(*z*)|_*z* = 1_ = 0 can be calculated, so *z* = 1 is stable. When *x* < *x*_2_,*y* < *y*_2_ or *p* > *p*_2_,*J*(*x, y, p*) < 0,$F^{\prime }\left(z\right){|}_{z=0}<0$ and *F*(*z*)|_*z* = 0_ = 0 can be calculated, so is stable. When *x* = *x*_2_, *y* = *y*_2_ or *p* = *p*_2_, *J*(*x, y, p*) = 0 and *F*′(*z*) = 0 can be calculated, we are unable to determine a stable strategy.

Proposition 3 shows that when *x*, *y*increases, or *p* decreases, the stability strategy of third-party testing agencies changes from collusion to non-collusion. Therefore, in the drug quality co-regulation supervision, the government should strictly restrict the behavior of third-party testing agencies and promote the work pattern of co-regulation supervision.

In summary, the response function of *z* is


(22) $$z=\{\begin{array}{ccc} 0 & if & x<1-\frac{y(V-C_{p})+(1-y)(F_{p}+C_{t}-B)}{(1-y)pF_{p}}\\ \left[0,1\right] & if & x=1-\frac{y(V-C_{p})+(1-y)(F_{p}+C_{t}-B)}{(1-y)pF_{p}}\\ 1 & if & x>1-\frac{y(V-C_{p})+(1-y)(F_{p}+C_{t}-B)}{(1-y)pF_{p}} \end{array}$$

**Figure 4:**
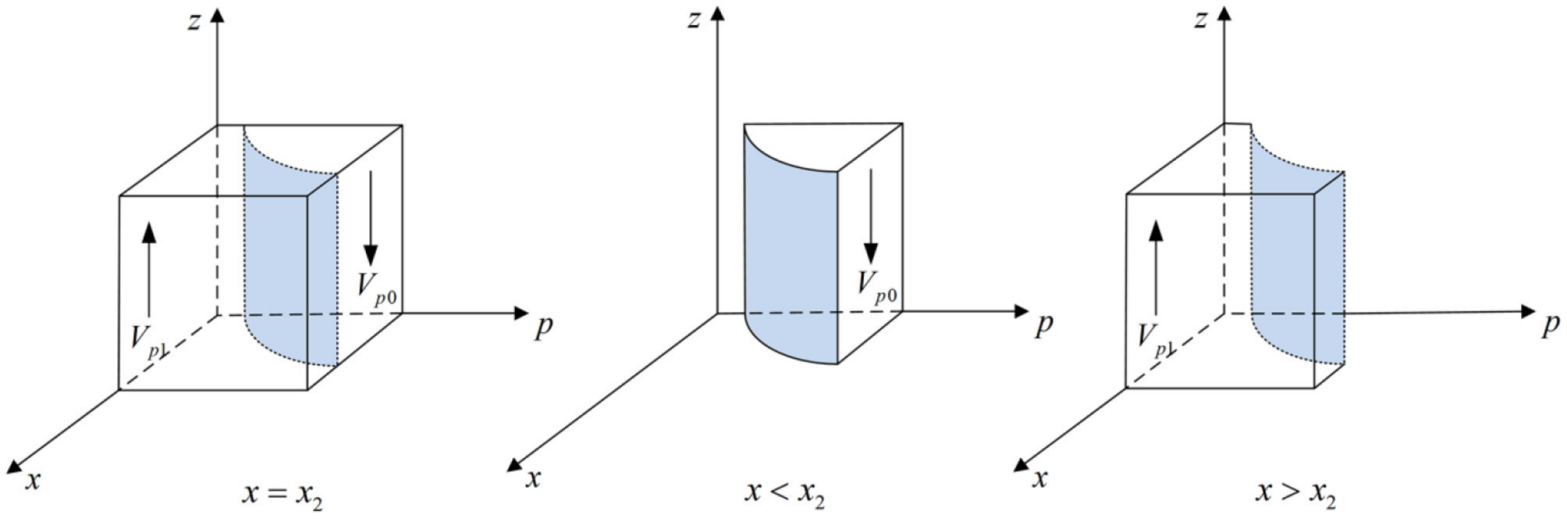
Phase diagram of third-party testing agencies strategic choice. Figure is the phase diagram that shows the evolutionary trend of third-party testing agencies' strategy obtained by calculating the response function of the probability of third-party testing agencies choosing the “non-collusion” strategy.

According to Proposition 3, the phase diagram of third-party testing agencies strategic choice is shown in Figure 4.

It can be seen from Figure 4 that the volume of *V*_*p*1_ is the probability that the third-party testing agencies choose the “non-collusion” strategy, and the volume of *V*_*p*0_ is the probability that the third-party testing agencies choose the “collusion” strategy. And,


(23) $$\begin{array}{l} V_{p0}=\int_{0}^{1}\int_{\frac{y\left(V-C_{p}\right)+\left(1-y\right)\left(F_{p}+C_{t}-B\right)}{\left(1-y\right)F_{p}}}^{1}x_{2}dpdz\\ =1-\frac{y\left(V-C_{p}\right)+\left(1-y\right)\left(F_{p}+C_{t}-B\right)}{\left(1-y\right)F_{p}}\\ \left[1+\ln\;\frac{\left(1-y\right)F_{p}}{y\left(V-C_{p}\right)+\left(1-y\right)\left(F_{p}+C_{t}-B\right)}\right] \end{array}$$

(24) $$\begin{array}{l} V_{p1}=1-V_{p0}{\frac{y\left(V-C_{p}\right)+\left(1-y\right)\left(F_{p}+C_{t}-B\right)}{\left(1-y\right)F_{p}}}^{1}x_{2}dpdz\\ =1-\frac{y\left(V-C_{p}\right)+\left(1-y\right)\left(F_{p}+C_{t}-B\right)}{\left(1-y\right)F_{p}}\\ \left[1+\ln\;\frac{\left(1-y\right)F_{p}}{y\left(V-C_{p}\right)+\left(1-y\right)\left(F_{p}+C_{t}-B\right)}\right] \end{array}$$

**Corollary 3.1** The probability of non-collusion by third-party testing agencies is positively related to the speculative cost of participating in collusion.

***Proof*** According to the probability, the first-order partial derivatives of can be calculated:

Corollary 3.1 shows that when the speculative cost of collusion by third-party testing agencies is higher, their motivation for collusion is weakened and the probability of choosing “non-collusion” is higher.

**Corollary 3.2** The probability of collusion by third-party testing agencies is positively related to the cost of drug enterprises seeking to collude.

***Proof*** According to the probability, the first-order partial derivatives of *B* can be calculated:


(25) $$\begin{array}{l} \frac{\partial V_{p0}}{\partial B}=\frac{1}{F_{p}}\\ \ln\;\frac{\left(1-y\right)F_{p}}{y\left(V-C_{p}\right)+\left(1-y\right)\left(F_{p}+C_{t}-B\right)}>0 \end{array}$$

Corollary 3.2 shows that when the cost of seeking collusion increases, in order to obtain economic benefits, the higher the probability that third-party testing agencies choose to “collusion.”

### New Media Strategic Choice Stability

The expected benefit of new media choosing the “true reporting” strategy is:


(26) $$\begin{array}{l} E_{p}=y\left(R_{ml}-C_{m}\right)+\left(1-y\right)\left(R_{mh}-C_{m}\right)+\left(1-y\right)\left(1-x\right)\\ \left(1-z\right)\left(R_{ml}-R_{mh}\right) \end{array}$$

The expected benefit of new media choosing the “distorted reporting” strategy is:


(27) $$\begin{array}{l} E_{1-p}=y\left(R_{mh}-C_{m}-F_{m}\right)+\left(1-y\right)\left(R_{mh}-C_{m}\right) \end{array}$$

The replicated dynamic equation and the first derivative of new media's strategic choice are:


(28) $$\begin{array}{l} F(p)=dp/dt=p(E_{p}-\bar{E})=p(1-p)(E_{p}-E_{1-p})\\ =p(1-p)[y(R_{ml}-R_{mh}+F_{m})+(1-y)(1-x)\\ \left(1-z)(R_{ml}-R_{mh})\right] \end{array}$$

(29) $$\begin{array}{l} F^{\prime }\left(p\right)=\left(1-2p\right)[y\left(R_{ml}-R_{mh}+F_{m}\right)+\left(1-y\right)\left(1-x\right)\\ \left(1-z\right)\left(R_{ml}-R_{mh}\right)] \end{array}$$

According to the stability theorem of differential equations, if the probability of new media choosing the “true reporting” strategy is to be in a stable state, the following conditions must be met: *F*(*p*) = 0 and *F*′(*p*) < 0.

**Proposition 3** When *x* > *x*_3_, *y* < *y*_3_ or *z* > *z*_3_, new media's stabilization strategy is “true reporting.” When, *y* > *y*_3_ or *z* < *z*_3_, its stabilization strategy is “distorted reporting.” When, *y* = *y*_3_ or *z* = *z*_3_, we are unable to determine its stabilization strategy. The thresholds are


$$\begin{array}{l} x_{3}=1-\frac{y\left(R_{ml}-R_{mh}+F_{m}\right)}{\left(1-y\right)\left(1-z\right)\left(R_{mh}-R_{ml}\right)},\\ y_{3}=\frac{\left(1-x\right)\left(1-z\right)\left(R_{ml}-R_{mh}\right)}{\left(1-x\right)\left(1-z\right)\left(R_{ml}-R_{mh}\right)-\left(R_{ml}-R_{mh}+F_{m}\right)},\\ z_{3}=1-\frac{y\left(R_{ml}-R_{mh}+F_{m}\right)}{\left(1-y\right)\left(1-x\right)\left(R_{mh}-R_{ml}\right)}. \end{array}$$

***Proof*** Make *H*(*x, y, z*) = *y*(*R*_*ml*_ − *R*_*mh*_ + *F*_*m*_) + (1 − *y*)(1 − *x*)(1 − *z*)(*R*_*ml*_ − *R*_*mh*_), when *H*(*x, y, z*) = 0„,$z_{3}=1-\frac{y\left(R_{ml}-R_{mh}+F_{m}\right)}{\left(1-y\right)\left(1-x\right)\left(R_{mh}-R_{ml}\right)}$ can be calculated. Because ∂*H*(*x, y, z*)/∂*x* > 0, ∂*H*(*x, y, z*)/∂*y* < 0 and ∂*H*(*x, y, z*)/∂*z* > 0, *H*(*x, y, z*) is an increasing function of *x* and *z*, and a decreasing function of *y*. When *x* > *x*_3_, *y* < *y*_3_ or *z* > *z*_3_ *H*(*x, y, z*) > 0,$F^{\prime }\left(p\right){|}_{p=1}<0$ and $F^{\prime }\left(p\right){|}_{p=1}=0$ can be calculated, so *p* = 1 is stable. When *x* < *x*_3_, *y* > *y*_3_ or, *H*(*x, y, z*) < 0,$F^{\prime }\left(p\right){|}_{p=0}<0$ and *F*(*p*)|_*p*=0_ = 0 can be calculated, so is stable. When *x* = *x*_3_, *y* = *y*_3_ or *z* = *z*_3_, *H*(*x, y, z*) = 0 and *F*′(*p*) = 0 can be calculated, we are unable to determine a stable strategy.

Proposition 4 shows that when*x*, *z* increases, or *y* decreases, the stability strategy of new media changes from distorted reporting to true reporting. Therefore, in drug quality supervision, new media can urge local government to strictly supervise, drug enterprises' integrity production, and third-party testing agencies to fulfill their social responsibility by reporting on drug quality information.

In summary, the response function of is:


(30) $$p=\{\begin{array}{ccc} 0 & if & x<1-\frac{y(R_{ml}-R_{mh}+F_{m})}{\left(1-y)(1-z)(R_{mh}-R_{ml}\right)}\\ \left[0,1\right] & if & x=1-\frac{y(R_{ml}-R_{mh}+F_{m})}{\left(1-y)(1-z)(R_{mh}-R_{ml}\right)}\\ 1 & if & x>1-\frac{y(R_{ml}-R_{mh}+F_{m})}{\left(1-y)(1-z)(R_{mh}-R_{ml}\right)} \end{array}$$

**Figure 5:**
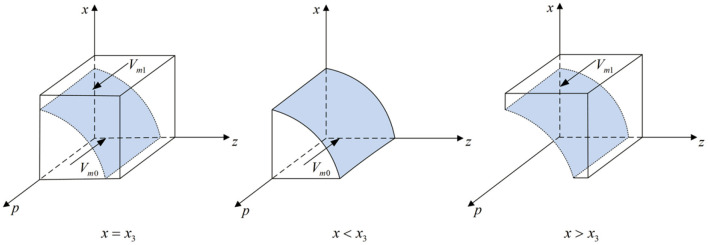
Phase diagram of new media strategic choice. Figure is the phase diagram that shows the evolutionary trend of new media's strategy obtained by calculating the response function of the probability of new media choosing the “true reporting” strategy.

According to Proposition 4, the phase diagram of new media strategic choice is shown in Figure 5.

It can be seen from Figure 5 that the volume of *V*_*m*1_ is the probability that new media chooses the “true reporting” strategy, and the volume of *V*_*m*0_is the probability that new media chooses the “distorted reporting” strategy. And,


(31) $$\begin{array}{l} V_{m0}=\int_{0}^{1}\int_{0}^{1-\frac{y\left(R_{ml}-R_{mh}+F_{m}\right)}{\left(1-y\right)\left(R_{mh}-R_{ml}\right)}}x_{3}dzdp\\ =1-\frac{y\left(R_{ml}-R_{mh}+F_{m}\right)}{\left(1-y\right)\left(R_{mh}-R_{ml}\right)}\left[1-\ln\;\frac{y\left(R_{ml}-R_{mh}+F_{m}\right)}{\left(1-y\right)\left(R_{mh}-R_{ml}\right)}\right] \end{array}$$

(32) $$\begin{array}{l} V_{m1}=1-V_{m0}=\frac{y\left(R_{ml}-R_{mh}+F_{m}\right)}{\left(1-y\right)\left(R_{mh}-R_{ml}\right)}\\ \left[1-\ln\;\frac{y\left(R_{ml}-R_{mh}+F_{m}\right)}{\left(1-y\right)\left(R_{mh}-R_{ml}\right)}\right] \end{array}$$

**Corollary 4.1** The probability of true reporting by new media is positively correlated with the compensation to drug enterprises.

***Proof*** According to the probability *V*_*m*1_, the first-order partial derivatives of can be calculated:


$$\begin{array}{l} \frac{\partial V_{m1}}{\partial F_{m}}=\frac{y}{\left(1-y\right)\left(R_{mh}-R_{ml}\right)}\ln\;\frac{\left(1-y\right)\left(R_{mh}-R_{ml}\right)}{y\left(R_{ml}-R_{mh}+F_{m}\right)}>0 \end{array}$$

Corollary 4.1 shows that as the cost of distorted reports increases, new media tend to report truthfully, which will help promote the social pattern of co-regulation supervision.

**Corollary 4.2** The probability of new media “distorted reporting” is positively correlated with the additional revenue gained from distorted reporting.

***Proof*** According to the probability *V*_*m*0_, the first-order partial derivatives of *R*_*mh*_ − *R*_*ml*_ can be calculated:


(33) $$\begin{array}{l} \frac{\partial V_{m0}}{\partial \left(R_{mh}-R_{ml}\right)}=\frac{F_{m}}{\left(1-y\right){\left(R_{mh}-R_{ml}\right)}^{2}}\\ \ln\;\frac{\left(1-y\right)\left(R_{mh}-R_{ml}\right)}{y\left(R_{ml}-R_{mh}+F_{m}\right)}>0 \end{array}$$

Corollary 4.2 shows that when the additional revenue of distorted reporting increases, new media will be more inclined to choose “distorted reporting.” Therefore, local government should pay attention to the construction of new media platform, strengthen the management and regulation of it, and build new media into a healthy and upward platform.

**Corollary 4.3** When *F*_*m*_ > *F*_*m*0_, new media will choose the “true reporting” strategy. When *F*_*m*_ < *F*_*m*0_, it will choose the “distorted reporting” strategy. The threshold is $F_{m0}=\frac{\left(R_{mh}-R_{ml}\right)\left[\left(1-y\right)\left(1-x\right)\left(1-z\right)+y\right]}{y}$.

***Proof*** According to Proposition 4, when *H*(*x, y, z*) = 0, can be calculated. Because ∂*H*(*x, y, z*)/∂*F*_*m*_ > 0, *H*(*x, y, z*) is an increasing function of *F*_*m*_. When *F*_*m*_ > *F*_*m*0_, *H*(*x, y, z*) > 0, $F^{\prime }\left(p\right){|}_{p=1}<0$ and *F*(*p*)|_*p* = 1_ = 0 can be calculated. When *F*_*m*_ < *F*_*m*0_, *H*(*x, y, z*) < 0, $F^{\prime }\left(p\right){|}_{p=0}<0$ and *F*(*p*)|_*p* = 0_ = 0 can be calculated.

Corollary 4.3 shows that when new media chooses to report distortedly, it needs to pay compensation to drug enterprises. If, it is possible to ensure that it will choose the “true reporting” strategy. Therefore, increasing compensation for distorted reporting can prompt new media to report truthfully, while at the same time making up for the losses caused by distorted reporting to drug enterprises, so as to avoid damaging the enthusiasm of them to produce high-quality drugs.

## Stability Analysis of Strategic Combination

The stability of each strategic combination can be judged according to *Lyapunov's* first *method*. If all the eigenvalues of the *Jacobian matrix* are negative, the equilibrium point is an evolutionary stable strategy (ESS). If at least one eigenvalue is positive, the equilibrium point is an unstable point. If the eigenvalues are all negative except for zero, the equilibrium point is in a critical state and the stability is uncertain. This article analyzes the stability of the 16 pure strategy Nash equilibrium points, the *Jacobian matrix* of the replication dynamic system is


$$\begin{array}{l} J=\left[\begin{array}{cccc} \partial F\left(x\right)/\partial x & \partial F\left(x\right)/\partial y & \partial F\left(x\right)/\partial z & \partial F\left(x\right)/\partial p\\ \partial F\left(y\right)/\partial x & \partial F\left(y\right)/\partial y & \partial F\left(y\right)/\partial z & \partial F\left(y\right)/\partial p\\ \partial F\left(z\right)/\partial x & \partial F\left(z\right)/\partial y & \partial F\left(z\right)/\partial z & \partial F\left(z\right)/\partial p\\ \partial F\left(p\right)/\partial x & \partial F\left(p\right)/\partial y & \partial F\left(p\right)/\partial z & \partial F\left(p\right)/\partial p \end{array}\right] \end{array}$$

### Stability of Strategic Combination in True Reporting

When the stability strategy of new media is “true reporting,” that is, when condition is satisfied, the asymptotic stability analysis of the equilibrium point of the replicated dynamic system is shown in Table 3.

**Table 3:** Asymptotic stability of the equilibrium point in true reporting.

**Equilibrium point**	**Eigenvalues λ_1_, λ_2_, λ_3_, λ_4_**	**Sign**	**Stability**
(0, 0, 1, 1)	*G*_*l*_ − *G*_*h*_ + *F*_*g*_,*C*_*l*_ − *C*_*h*_ + *F*_*e*_ + δ(*N* + *I*),*B* − *C*_*t*_, 0	(×, +, ×, 0)	Unstable
(1, 0, 1, 1)	*G*_*h*_ − *G*_*l*_ − *F*_*g*_,*C*_*l*_ − *C*_*h*_ + *F*_*e*_ + δ(*N* + *I*),*B* − *C*_*t*_ − *F*_*p*_, 0	(×, +, ×, 0)	Unstable
(0, 1, 1, 1)	*G*_*l*_ − *G*_*h*_,−[*C*_*l*_ − *C*_*h*_ + *F*_*e*_ + δ(*N* + *I*)],*C*_*p*_ − *V*,*R*_*mh*_ − *R*_*ml*_ − *F*_*m*_	(−, −, ×, −)	When condition is satisfied, it is ESS
(0, 0, 0, 1)	*G*_*l*_ − *G*_*h*_ + *F*_*e*_ + *F*_*p*_,*C*_*l*_ − *C*_*h*_ + *B*,*C*_*t*_ − *B*,*R*_*mh*_ − *R*_*ml*_	(×, ×, ×, +)	Unstable
(1, 1, 0, 1)	*G*_*h*_ − *G*_*l*_,−[*C*_*l*_ − *C*_*h*_ + *B* + *F*_*e*_ + δ(*N* + *I*)],*V* − *C*_*p*_,	(+, −, ×, −)	Unstable
(1, 0, 0, 1)	*G*_*h*_ − *G*_*l*_ − *F*_*e*_ − *F*_*p*_,*C*_*l*_ − *C*_*h*_ + *B* + *F*_*e*_ + δ(*N* + *I*),*F*_*p*_ + *C*_*t*_ − *B*, 0	(×, +, ×, 0)	Unstable
(0, 1, 0, 1)	*G*_*l*_ − *G*_*h*_,*C*_*h*_ − *C*_*l*_ + *B*,*V* − *C*_*p*_,*R*_*mh*_ − *R*_*ml*_ − *F*_*m*_	(−, +, ×, −)	Unstable
(1, 1, 1, 1)	*G*_*h*_ − *G*_*l*_,−[*C*_*l*_ − *C*_*h*_ + *F*_*e*_ + δ(*N* + *I*)],*C*_*p*_ − *V*,*R*_*mh*_ − *R*_*ml*_ − *F*_*m*_	(+, −, −, −)	Unstable

*Indicates that the sign is uncertain. If the condition is met, it is, respectively, stable point. Condition : C_p_ < V*.

It can be seen from Table 3 that if the new media reports truthfully, when *C*_*p*_ < *V*, the equilibrium point (0, 1, 1, 1) of the replicated dynamic system is ESS.

The production of high-quality drugs by drug enterprises, non-collusion by third-party testing agencies, and true reporting by new media are the most ideal situation for local government. Therefore, when drug enterprises produce low-quality drug, local government must actively perform its supervision responsibility and impose severe penalty on them; encourage new media to report truthfully in a timely manner, so as to urge drug enterprises consciously produce high-quality drug.

### Stability of Strategic Combination in Distorted Reporting

When the stability strategy of new media is “distorted reporting,” that is, when condition *R*_*mh*_ − *R*_*ml*_ > *F*_*m*_ is satisfied, the asymptotic stability analysis of the equilibrium point of the replicated dynamic system is shown in Table 4.

**Table 4:** Asymptotic stability of the equilibrium point in distorted reporting.

**Equilibrium point**	**Eigenvalues λ_1_, λ_2_, λ_3_, λ_4_**	**Sign**	**Stability**
(0, 0, 1, 0)	*G*_*l*_ − *G*_*h*_ + *F*_*g*_,*C*_*l*_ − *C*_*h*_ + *F*_*m*_ + *F*_*e*_ + δ(*N* − *L*),*B* − *C*_*t*_ − *F*_*p*_, 0	(×, +, ×, 0)	Unstable
(1, 0, 1, 0)	*G*_*h*_ − *G*_*l*_ − *F*_*g*_, *C*_*l*_ − *C*_*h*_ + *F*_*m*_ + *F*_*e*_ + δ(*N* − *L*),*B* − *C*_*t*_ − *F*_*p*_, 0	(×, +, ×, 0)	Unstable
(0, 1, 1, 0)	*G*_*l*_ − *G*_*h*_,−[*C*_*l*_ − *C*_*h*_ + *F*_*m*_ + *F*_*e*_ + δ(*N* − *L*)],*C*_*p*_ − *V*,*R*_*ml*_ − *R*_*mh*_ + *F*_*m*_	(−, −, ×, −)	When condition ② is satisfied, it is ESS
(1, 1, 1, 0)	*G*_*h*_ − *G*_*l*_ − *F*_*g*_,−[*C*_*l*_ − *C*_*h*_ + *F*_*m*_ + *F*_*e*_ + δ(*N* − *L*)],*C*_*p*_ − *VR*_*ml*_ − *R*_*mh*_ + *F*_*m*_	(×, −, ×, −)	When condition ③ is satisfied, it is ESS
(1, 1, 0, 0)	*G*_*h*_ − *G*_*l*_,−[*C*_*l*_ − *C*_*h*_ + *F*_*m*_ + *B* + *F*_*e*_ + δ(*N* − *L*)],*V* − *C*_*p*_,*R*_*ml*_ − *R*_*mh*_ + *F*_*m*_	(+, −, ×, −)	Unstable
(1, 0, 0, 0)	*G*_*h*_ − *G*_*l*_ − *F*_*g*_,*C*_*l*_ − *C*_*h*_ + *F*_*m*_ + *B* + *F*_*e*_ + δ(*N* − *L*),*F*_*p*_ + *C*_*t*_ − *B*,	(×, +, ×, 0)	Unstable
(0, 1, 0, 0)	*G*_*l*_ − *G*_*h*_,−[*C*_*l*_ − *C*_*h*_ + *F*_*m*_ + *B* + *F*_*e*_ + δ(*N* − *L*)],*V* − *C*_*p*_,*R*_*ml*_ − *R*_*mh*_ + *F*_*m*_	(−, −, ×, −)	When condition ④ is satisfied, it is ESS
(0, 0, 0, 0)	*G*_*l*_ − *G*_*h*_ + *F*_*g*_,*C*_*l*_ − *C*_*h*_ + *F*_*m*_ + *B* + *F*_*e*_ + δ(*N* − *L*),*F*_*p*_ + *C*_*t*_ − *B*,*R*_*ml*_ − *R*_*mh*_	(×, +, ×, −)	Unstable

*× Indicates that the sign is uncertain. If the conditions ②, ③ and ④ are met, they are, respectively, stable points. Condition ②: C_p_ < V; Condition ③:G_h_ − G_l_ < F_g_,C_p_ < V; Condition ④:C_p_ > V*.

It can be seen from Table 4 that if the new media reports distortedly, when *C*_*p*_ < *V*, the equilibrium point (0, 1, 1, 0) of the replicated dynamic system is ESS. When *G*_*h*_ − *G*_*l*_ < *F*_*g*_ and *C*_*p*_ < *V*, the equilibrium point (1, 1, 1, 0) of the replicated dynamic system is ESS. When *C*_*p*_ > *V*, the equilibrium point (0, 1, 0, 0) of the replicated dynamic system is ESS.

When drug enterprises produce high-quality drug, in order to obtain economic benefits, new media will have distorted reporting, which will undermine the enthusiasm of drug enterprises to produce high-quality drug. Therefore, it is necessary to encourage all parties to perform their social responsibilities and play their respective roles. Standardize the behavior of new media and impose legal sanctions on distortions, rumors and so on. In addition, it is necessary to guide it to build a positive platform to better reflect the authority of the co-regulation information platform.

## Simulation Analysis

This article uses *Matlab 2020b* for numerical simulation to verify the validity of the evolutionary stability analysis. Based on the actual conditions and the research of other scholars, this article makes assumptions about the numerical values in Table 5.

**Table 5:** Simulation numerical assumptions.

*G*_*h*_ − *G*_*l*_ = 7	*C*_*h*_ − *C*_*l*_ = 7	*R*_*mh*_ = 5	*N* = 3	*C*_*t*_ = 1	= 2.5
*F*_*g*_ = 3	*R*_*e*_ = 15	*F*_*m*_ = 3	*C*_*p*_ = 2	*F*_*p*_ = 3	*L* = 4
*B* = 4	*F*_*e*_ = 4	= 0.5	*I* = 2	*V* = 4	*R*_*ml*_ = 3

### Impact of Reputation Value and Loss

**Figure 6:**
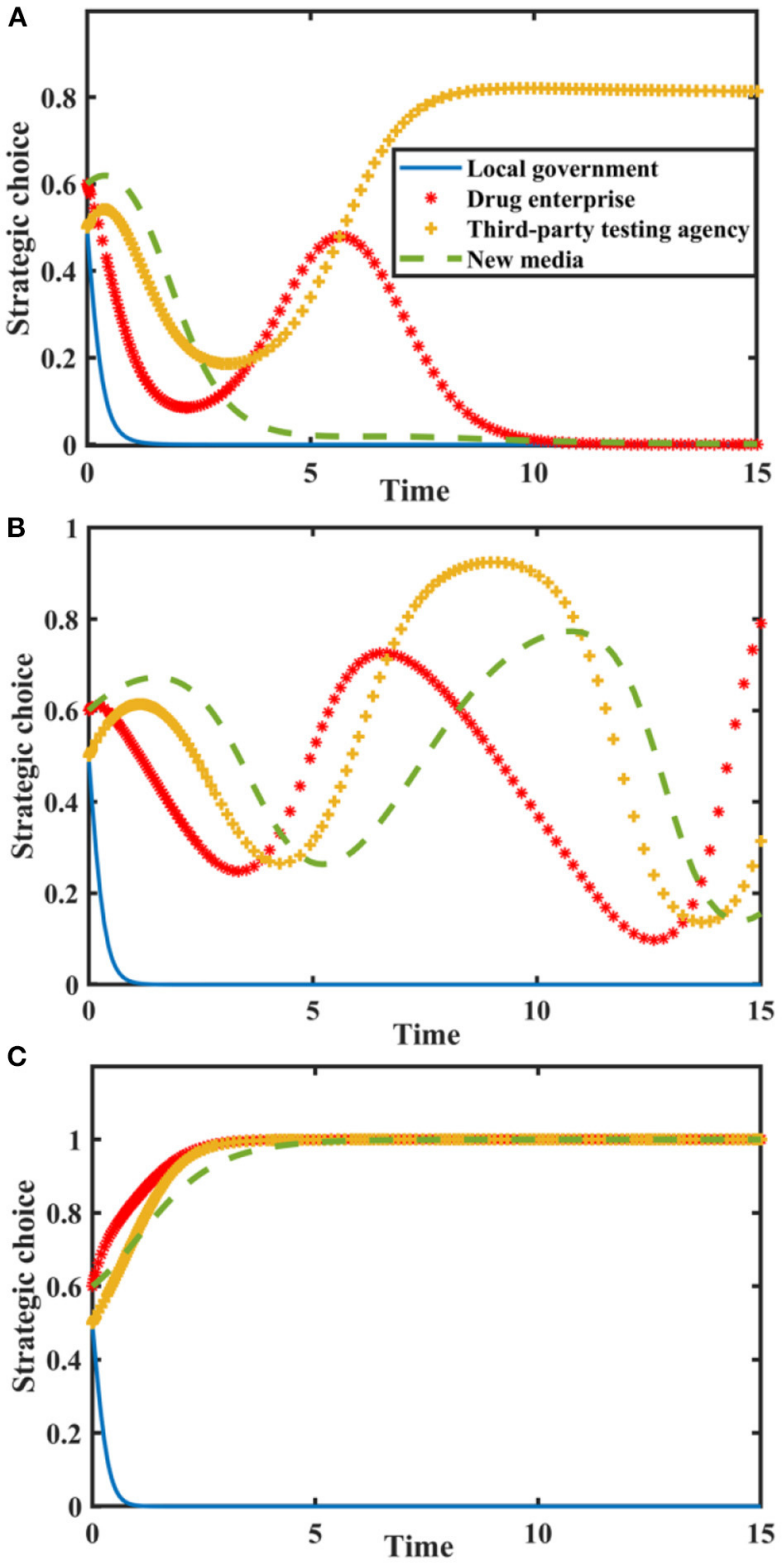
Impact of *I* and *N* on the evolution of each player's strategy. Figure is the simulation diagram that shows the influence of the reputation value brought by new media true reporting and reputation loss brought by new media distorted reporting on the strategic choices of local government, drug enterprises, third-party testing agencies, and new media. **(A)** When the situation *I* = 0.0, *N* = 0.0. **(B)** When the situation *I* = 2.0, *N* = 3.0. **(C)** When the situation *I* = 4.0, *N* = 6.0.

Set *I* = {0.0, 2.0, 4.0}, *N* = {0.0, 3.0, 6.0}, and the evolution process and results of players' strategy of the quartet game are shown in Figure 6.

Figure 6 shows that when *I* and *N* are zero, the stable strategy of drug enterprises is to produce low-quality drugs. At this time, local government chooses loose supervision, new media chooses distorted reporting, and the probability of third-party testing agencies choosing non-collusion is stable at a relatively high level. With the increase of and, local government's stable strategy is loose supervision, the strategic choices of others continue to fluctuate. When *I* and *N* further increase, drug enterprises produce high-quality drug, new media reports truthfully, and third-party testing agencies are not colluding. At this time, local government doesn't need to conduct strict supervision. New media must actively play a supervisory and reporting role, urge drug enterprises to produce high-quality drugs, and establish a good image. Therefore, local government should improve drug quality public opinion supervision mechanism, use the co-regulation information platform to share information with new media, and give full play to the advantages of new media in drug quality supervision.

### Impact of Influence Effect and Additional Revenue From Distorted Reporting

**Figure 7:**
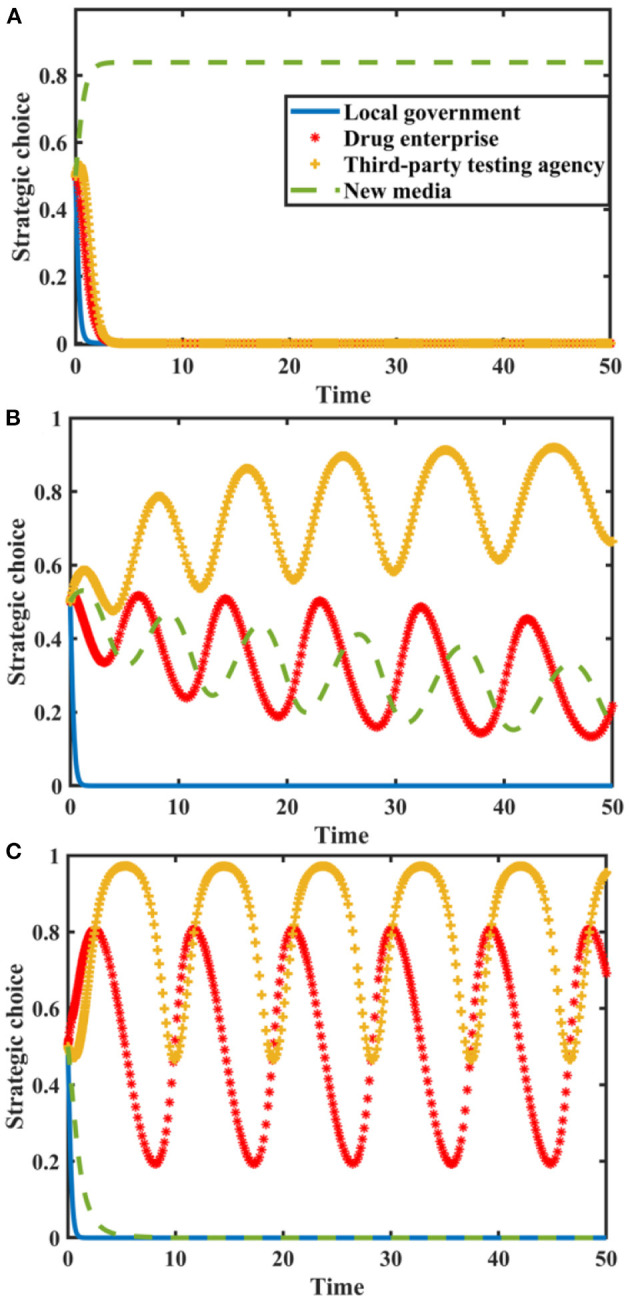
Impact of δ and on the evolution of each player's strategy. Figure is the simulation diagram that shows the influence of the influence effect of new media reports and additional revenue from distorted reporting on the strategic choices of local government, drug enterprises, third-party testing agencies, and new media. **(A)** When the situation δ = 0.0, *R*_*mh*_ − *R*_*ml*_ = 0.0, **(B)** When the situation δ = 0.4, *R*_*mh*_ − *R*_*ml*_ = 2.0. **(C)** When the situation δ = 0.8, *R*_*mh*_ − *R*_*ml*_ = 4.0.

Set δ = {0.0, 0.4, 0.8}, *R*_*mh*_ − *R*_*ml*_ = {0.0, 2.0, 4.0}, and the evolution process and results of players' strategy of the quartet game are shown in Figure 7.

Figure 7 shows that when and *R*_*mh*_ − *R*_*ml*_ are zero, the stability strategy of third-party testing agencies is collusion, the stability strategy of drug enterprises is the producing low-quality drugs, and the stability strategy of local government is loose supervision. The probability of new media choosing true reporting strategy is stable at a higher probability. When δ and *R*_*mh*_ − *R*_*ml*_ increase, local government loosely supervises, and the strategic choices of drug enterprises, third-party testing agencies and new media are in a state of fluctuation. With the further increase in δ and, the strategic choices of drug enterprises and third-party testing agencies are in a state of fluctuation, local government loosely supervises, and new media distortedly reports. Therefore, we must pay attention to the construction of new media platforms, standardize the dissemination of drug quality information, and realize the active governance function of new media.

### Impact of Additional Cost

**Figure 8:**
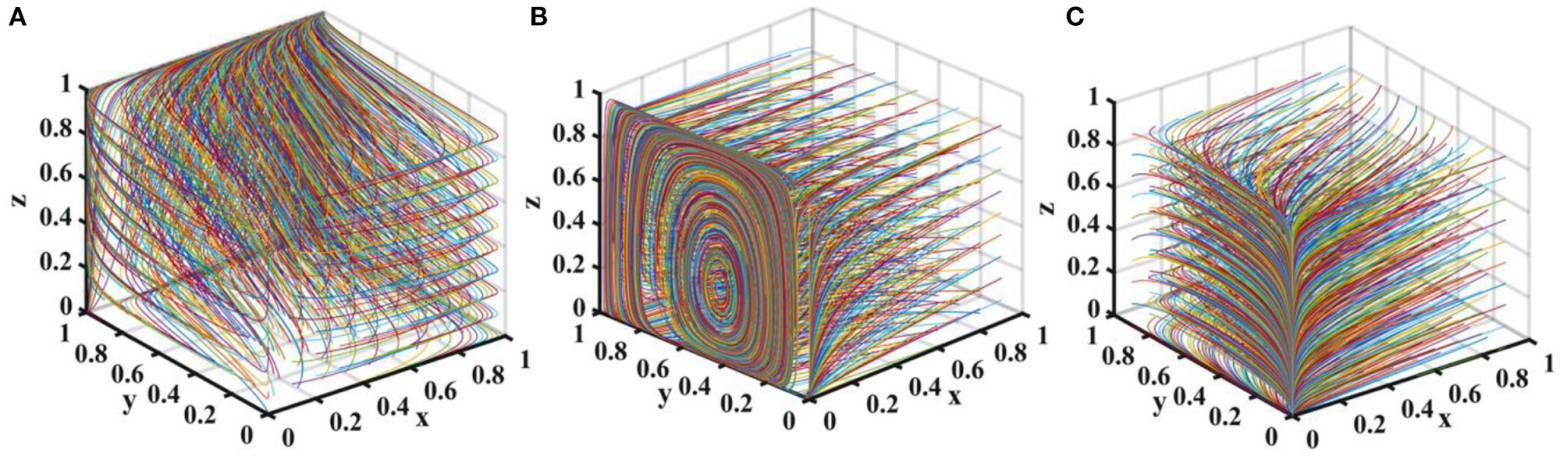
Impact of additional costs on the evolution of each player's strategy. Figure is the simulation diagram that shows the influence of the additional costs on the strategic choices of local government, drug enterprises, and third-party testing agencies. **(A)** When the situation *G*_*k*_ − *G*_*l*_ = 0.0, *C*_*k*_ − *C*_*l*_ = 0.0. **(B)** When the situation *G*_*k*_ − *G*_*l*_ = 7.0, *C*_*k*_ − *C*_*l*_ = 7.0. **(C)** When the situation *G*_*k*_ − *G*_*l*_ = 11.0, *C*_*k*_ − *C*_*l*_ = 11.0.

Let the probability that new media chooses true reporting is *p* = 0.5. Suppose *G*_*h*_ − *G*_*l*_ = {0.0, 7.0, 11} and *C*_*h*_ − *C*_*l*_ = {0.0, 7.0, 11}. The tripartite game evolutionary process and results of local government, drug enterprises, and third-party testing agencies are shown in Figure 8.

It can be seen from Figure 8 that when the additional costs are zero, there is an evolutionary stable equilibrium point (1, 1, 1) in the replication dynamic system. At this time, the drug enterprises produce high-quality drugs, third-party testing agencies don't participate in collusion, and the local government chooses strict supervision. As the additional costs increase, the replication dynamic system is in an unstable state; when the additional costs further increase, the evolutionary stable equilibrium point of the replication dynamic system is (0, 0, 0). Therefore, in order to maintain a good market order, the central government should reduce the cost of strict supervision by local government and improve its supervision efficiency. Reduce the burden on drug enterprises to produce high-quality drugs, make good use of the co-regulation information platform, and promote a good situation in co-regulation supervision.

### Impact of Penalty

**Figure 9:**
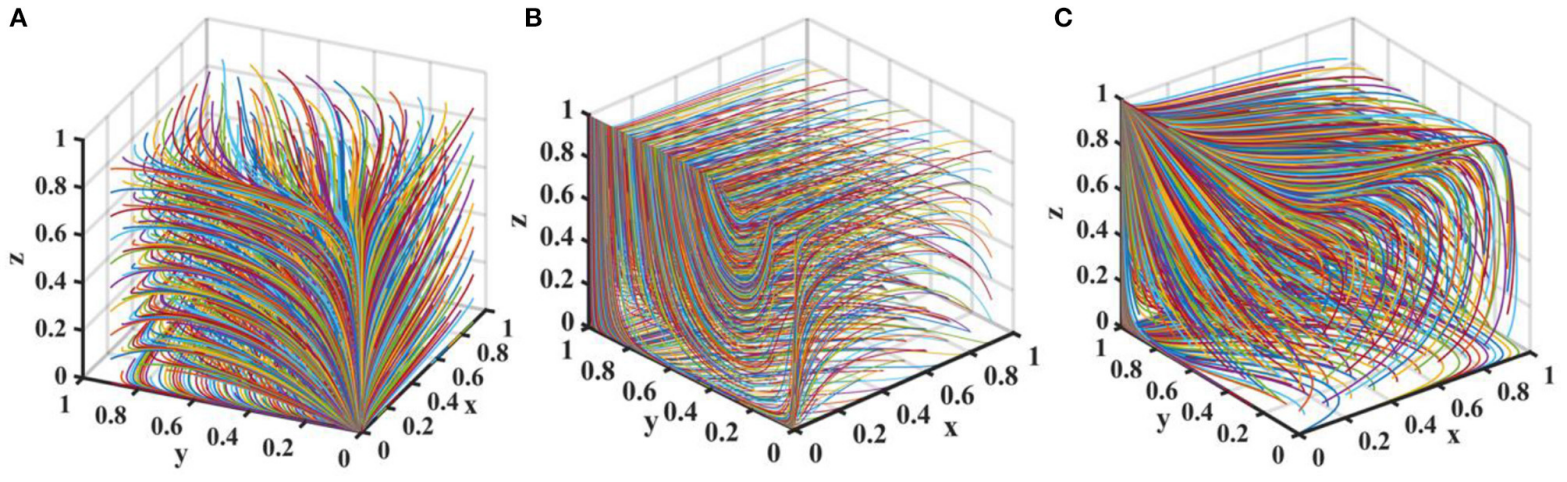
Impact of penalties on the evolution of each player's strategy. Figure is the simulation diagram that shows the influence of the penalties on the strategic choices of local government, drug enterprises, and third-party testing agencies. **(A)** When the situation *F*_*g*_ = 0.0, *F*_*e*_ = 0.0. **(B)** When the situation *F*_*g*_ = 4.5, *F*_*e*_ = 4.5. **(C)** When the situation *F*_*g*_ = 6.0, *F*_*e*_ = 6.0.

Suppose the probability of new media true reporting is *p* = 0.5. Let *F*_*g*_ = {0.0, 4.5, 6.0}, *F*_*e*_ = {0.0, 4.5, 6.0}, and *F*_*p*_ = {0.0, 4.5, 6.0}. The tripartite game evolutionary process and results of local government, drug enterprises, and third-party testing agencies are shown in Figure 9.

It can be seen from Figure 9 that when there is no penalty, the evolutionary stable equilibrium point is (0, 0, 0). As the penalties increase, the replication dynamic system is in an unstable state. When the penalty is further increased, the equilibrium point evolves to (0, 1, 1). At this time, the local government is aware of this and will conduct loose supervision. Therefore, penalty has a restrictive effect on the strategic choice of drug enterprises, but it's not conducive to the performance of strict supervision duty by local government. It's necessary to regulate the behavior of third-party testing agencies, give full play to their supervisory role, and urge drug enterprises to produce high-quality drugs in strict accordance with production standards.

## Discussions

Considering new media participation and third-party testing agencies' collusion behavior, this article constructs a quartet evolutionary game model. This article analyzes the influence of the change of each decision variable on the strategy evolution and puts forward the following suggestions.

First of all, complete a co-regulation information platform, promote the establishment of a good co-regulation supervision system, and emphasize the joint participation of local government, drug enterprises, new media, and third-party testing agencies. It is necessary to mobilize the forces of all sectors of society to promote the formation of a drug quality supervision pattern involving government supervision, drug enterprises operation, new media reporting, and third-party testing agencies participation.

Secondly, fully implement the main responsibility of drug enterprises, establish a “blacklist” system for them, notify and criticize enterprises that produce low-quality drugs, increase the scale and frequency of drug sampling, and give play to their self-discipline capability. In addition, drug enterprises should actively use new media platform to release authoritative information, track drug quality events and provide scientific professional knowledge explanations. Improve the public's ability to discriminate, and increase interactive communication through platforms such as Facebook, Twitter, and WeChat. Guide the public to use drug safely, and avoid losses caused by negative effects.

Besides, we must attach importance to the construction of new media platforms, strengthen the management of new media, and impose legal sanctions on unscrupulous media that distortedly reports. Standardize the dissemination of drug quality information, improve the quality of new media practitioners, and build it into a healthy and upward platform. Give full play to the assisting role of media supervision to government supervision, further win the trust of the public, and realize the positive governance function of new media.

Eventually, standardize the behavior of third-party testing agencies. Encourage them to fulfill their social responsibilities, feedback real drug quality test reports to the co-regulation information platform, and promote a good situation in drug quality co-regulation supervision.

## Conclusions

The sharing and interactive nature of new media makes it play an important role in drug quality supervision. Promoting the modernization of drug quality supervision requires the joint participation of multiple parties. Improve the social responsibility awareness of drug enterprises, increase policy support, reduce their production costs, and encourage them to consciously produce high-quality drugs. Restrict the behavior of third-party testing agencies, urge them not to participate in collusion, and assist local government to strictly supervise drug enterprises. Give full play to the advantages and functions of new media and encourage it to actively participate in co-regulation. According to the information provided by the co-regulation information platform, true reporting by new media are of great significance to enhancing the dissemination, guidance, and credibility of local government news releases.

Considering the strategic choices of local government, drug enterprises, third-party testing agencies, and new media, this article constructs a four-party evolutionary game model. However, the constructed game model is fully informative and single-stage under bounded rationality, and the game sequence is not considered. Therefore, considering the condition of incomplete information, constructing a multi-stage, repetitive and dynamic game model with multi-agent participation is the next research direction.

## Data Availability Statement

The original contributions presented in the study are included in the article/supplementary material, further inquiries can be directed to the corresponding author/s.

## Author Contributions

SZ wrote the manuscript and analyzed the data. LZ revised the manuscript and solved the model. All authors contributed to the article and approved the submitted version.

## Funding

This work was supported by the National Social Science Fund of China under grant Nos. 20BGL272 and 21ZDA024.

## Conflict of Interest

The authors declare that the research was conducted in the absence of any commercial or financial relationships that could be construed as a potential conflict of interest.

## Publisher's Note

All claims expressed in this article are solely those of the authors and do not necessarily represent those of their affiliated organizations, or those of the publisher, the editors and the reviewers. Any product that may be evaluated in this article, or claim that may be made by its manufacturer, is not guaranteed or endorsed by the publisher.
